# Improvements in readiness to change and drinking in primary care patients with unhealthy alcohol use: a prospective study

**DOI:** 10.1186/1471-2458-9-101

**Published:** 2009-04-09

**Authors:** Nicolas Bertholet, Nicholas J Horton, Richard Saitz

**Affiliations:** 1 Clinical Addiction Research and Education (CARE) Unit, Section of General Internal Medicine, Boston Medical Center, Boston University School of Medicine, Boston, Massachusetts, USA; 2 Clinical Epidemiology Center, Institute of Social and Preventive Medicine, Centre Hospitalier Universitaire Vaudois, University of Lausanne, Lausanne, Switzerland; 3 Department of Mathematics and Statistics, Smith College, Northampton, Massachusetts, USA; 4 Youth Alcohol Prevention Center, Boston University School of Public Health, Boston, Massachusetts, USA; 5 Department of Epidemiology, Boston University School of Public Health, Boston, Massachusetts, USA

## Abstract

**Background:**

The course of alcohol consumption and cognitive dimensions of behavior change (readiness to change, importance of changing and confidence in ability to change) in primary care patients are not well described. The objective of the study was to determine changes in readiness, importance and confidence after a primary care visit, and 6-month improvements in both drinking and cognitive dimensions of behavior change, in patients with unhealthy alcohol use.

**Methods:**

Prospective cohort study of patients with unhealthy alcohol use visiting primary care physicians, with repeated assessments of readiness, importance, and confidence (visual analogue scale (VAS), score range 1–10 points). Improvements 6 months later were defined as no unhealthy alcohol use or any increase in readiness, importance, or confidence. Regression models accounted for clustering by physician and adjusted for demographics, alcohol consumption and related problems, and discussion with the physician about alcohol.

**Results:**

From before to immediately after the primary care physician visit, patients (n = 173) had increases in readiness (mean +1.0 point), importance (+0.2), and confidence (+0.5) (all p < 0.002). In adjusted models, discussion with the physician about alcohol was associated with increased readiness (+0.8, p = 0.04). At 6 months, many participants had improvements in drinking or readiness (62%), drinking or importance (58%), or drinking or confidence (56%).

**Conclusion:**

Readiness, importance and confidence improve in many patients with unhealthy alcohol use immediately after a primary care visit. Six months after a visit, most patients have improvements in either drinking or these cognitive dimensions of behavior change.

## Background

Unhealthy alcohol use (the spectrum from at-risk drinking amounts through alcohol dependence) and its consequences represent a major burden of disease in the general population [1,2]. Among those with unhealthy alcohol use, brief intervention (BI) and motivational interviewing have demonstrated evidence of efficacy [3-5]. In primary care, BI is recommended by national practice guidelines (US Preventive Services Task Force, 2004), and, as part of BI, clinicians are encouraged to assess motivation and readiness to change, and to help patients increase readiness [6]. These changes in readiness are seen as short term goals on the way to decreased consumption [7,8].

Processes of change have been conceptualized in various ways; in the Transtheoretical model, Prochaska and DiClemente described the progression of individuals through stages of change [9]. Others have pointed out the role of importance of change for the patient [10], while Bandura emphasized the role of self efficacy, or confidence in ability to change [11]. Importance and confidence are considered components of readiness or readiness-related factors. For the purpose of this article we will refer to readiness, importance and confidence as 3 behavior change constructs, cognitive dimensions of behavior change, that comprise "readiness to change."

These three constructs can facilitate clinical conversations about health behaviors, and may even have predictive value for later behavior change [12-14]. They can be assessed with visual analog scales (VAS), which, because of their brevity, can be widely used. Longer assessments such as the Readiness to Change Questionnaire (RTCQ) (developed to assess readiness per se, not importance or confidence specifically) allow classification according to stage of readiness to change in addition to the computation of a continuous score [15].

The predictive value of cognitive dimensions of behavior change, however, has been mixed. Demmel et al. demonstrated that in alcohol dependent inpatients, readiness accounted for 9.4% of the variance in outcome [12]. Self-efficacy also appears to be a predictor of abstinence [16]. In other studies, however, readiness was not associated with subsequent consumption, and was predictive of more, not fewer consequences (or at least greater recognition of consequences) [17,18].

Thus, how behavior change constructs relate to later behavior change is not well understood. Furthermore, how these constructs change over time and in response to brief counseling, particularly in general healthcare settings, is also not well known. The efficacy for BI in primary care for unhealthy alcohol use is modest, and interventions with different theoretical rationales (e.g. MATCH and COMBINE studies) lead to similar results [10,19,20], calling into question the roles played by behavior change versus other constructs. These concerns are also encountered more broadly in psychotherapy research, and were named by Rosenzweig in 1936 after Alice in Wonderland as the "Dodo Bird effect" [21]. Rosenzweig proposed that "common" factors were responsible for the efficacy of psychotherapy. He used Lewis Carroll's Alice in Wonderland's Dodo bird conclusion "everybody has won and all must have prizes." In most trials of alcohol BI, control (and intervention) groups decrease consumption over time (one way to understand natural history in these settings). This improvement has a number of possible explanations: regression to the mean, effects of assessments or contact with study staff, natural history, and selection of individuals more prone to change (since agreement to participate might indicate some desire to change) [22]. A better understanding of behavior change constructs, the natural history of behavior change, and how the constructs relate to outcome might therefore inform the design of more efficacious interventions that achieve and sustain changes in drinking [23]. Therefore we studied improvements and predictors of improvements in 3 behavior change constructs (readiness, importance, confidence) and subsequent drinking after a primary care visit in a prospective cohort of patients.

## Methods

We studied a prospective cohort of adults with unhealthy alcohol use visiting an urban academic primary care practice who participated in a randomized trial of the impact of providing (or not providing) primary care physicians with patients' alcohol screening results [24]; physicians were the unit of randomization. Additional detail regarding the clinical trial has been published [24]. Patients were screened if they had a visit with an included physician. A trained staff researcher approached and interviewed enrolled subjects before and after their visits with a physician (February 1998–August 1999) in the waiting room. Patients were told they were being asked (initial screening) questions for research purposes. Eligible patients were told they were being asked to participate because they were primary care patients who reported drinking alcohol, and that the study might help physicians learn how to identify alcohol use by patients. Six months later, subjects were interviewed by telephone, after which they received compensation in the form of a voucher worth ten U.S. dollars. The screening was done using the CAGE questionnaire and the 3 questions published in the 1995 NIAAA guide to assess quantity and frequency of drinking [25,26]. The CAGE and the 3 alcohol questions were completed by paper and pencil in the waiting room or by help of a research assistant when needed. The baseline assessment was done by face to face interview. Inclusion for the present study was based on drinking as assessed using the Timeline Followback method [27]. Subjects were asked how many drinks they had each day for the past 30 days using a calendar and chronologic cues (e.g. weekends, holidays, significant events during the time period) based on published instructions for this assessment.

For the present study, eligible patients drank risky amounts (> 14 U.S. standard drinks [12 g each]/week or > 4 drinks/occasion for men and > 7 drinks/week or > 3 drinks/occasion for women in the past month), were fluent in English or Spanish, and had data available on 3 behavior change constructs of interest. Subjects were interviewed before, immediately after the visit and 6 months later. At each time point, subjects completed 3 visual analog scales (VASs), one for each of 3 behavior constructs ("how ready are you to change your drinking habits", "how important is it for you right now to change your drinking", "if you decide to change your drinking habits, how confident are you that you would succeed", with a score of 1 being "not ready/not important to change/not confident to succeed" and 10 "ready/very important to change/very confident to succeed") [17,28]. Subjects also completed the RTCQ, a validated instrument that assesses readiness that has satisfactory test-retest reliability and consists of 12 statements, each one evaluated by the participant on a 5 point Likert scale [29].

For the present study, we used a continuous RTCQ score (-24 to +24) that has good reliability (alpha = 0.85–0.86)[30,31]. The readiness item was included in the screener (paper and pencil). Confidence and importance items and the RTCQ were included in the previsit interview (face to face interview). After the visit and 6 months later, subjects completed the 30-day Timeline Followback, a validated calendar method considered a reference standard for assessing alcohol consumption,[32] and the Short Inventory of Problems (SIP) to assess alcohol-related problems [33]. Immediately after the visit, subjects were asked by research assistants if they had had any discussion with their physician about alcohol consumption as well as the content of such a discussion (i.e. "Did the doctor give you advice about your drinking habits," "Did the doctor talk about drinking?", "Did the doctor tell you how many drinks would be safe for you to drink?", "Did the doctor recommend that you cut down/quit drinking, go to Alcoholics Anonymous/treatment?"). Research assistants also assessed demographics, social support ("Do you currently have a partner?"), and illicit drug use over the past month ("In the last 30 days have you used marijuana/cocaine/heroin/other illegal drug?"). The study was approved by the Boston University Medical Center Institutional Review Board and a Certificate of Confidentiality was obtained from the U.S. government.

Analyses were performed using SAS software 9.1.3 (Cary, North Carolina). P values less than 0.05 were considered to be statistically significant. All analyses controlled for clustering of subjects within physician and physician randomization group, using Generalized Estimating Equations (GEE) regression models with an exchangeable working correlation and empirical variance estimator. Of note, in the original trial, the intervention (i.e. randomization group) was not associated with improvements in drinking risky amounts.

We assessed the outcomes changes in readiness, importance, and confidence between pre- and immediate post-visit assessments. Differences were computed by subtracting the pre-visit score from the immediate post-visit score. We used unadjusted models (but accounting for clustering) and models that adjusted for predictors to assess the changes between pre- and immediate post-visit assessments in each of the behavior change constructs.

Because a change in behavior could take place during the 6-month follow-up period after the pre-visit assessment, the state of readiness to change drinking at 6 months could become irrelevant (e.g. readiness to change in a subject who no longer consumed alcohol). Therefore, to assess change outcomes, we created 3 dichotomous variables representing "improvement," each defined as either no longer drinking risky amounts or, if still drinking risky amounts, having improvement (difference > 0) in readiness to change drinking (i.e. improvement in drinking *or *readiness), importance of changing drinking (i.e. improvement in drinking *or *importance), or confidence in ability to change drinking (i.e. improvement in drinking *or *confidence). Since readiness, importance and confidence are often considered intermediate outcomes, it is of clinical relevance to use an outcome taking into account increases in these readiness to change constructs [34,35]. We did not analyze these behavior change constructs in those who had already changed, and we did not have data on readiness/importance/confidence to sustain changes in drinking (e.g. not drinking risky amounts), and therefore could not analyze these constructs as outcomes.

Predictors of interest were: demographics (age, sex, race/ethnicity), social support (currently having a partner), alcohol consumption at study entry (drinks per day), SIP score, illicit drug use (marijuana, heroin, cocaine, other), and discussion with the physician about alcohol consumption during the visit.

All analyses were repeated with the RTCQ continuous score as the outcome, in order to corroborate the results obtained with the readiness VAS.

## Results

Of 4143 patients approached, 182 did not complete the screener. Of 487 who reported drinking risky amounts in the past month, 235 refused participation in the study and 18 had no time before the visit to complete the pre-visit assessment. Of the 234 remaining enrolled patients, our analytic sample was restricted to the 173 subjects drinking risky amounts who had available data on "readiness to change" at all 3 assessments (Table 1); they saw one of 36 physicians. We tested potential differences between included subjects who completed and did not complete the follow up: There were no significant differences (p < 0.05) between study subjects (n = 173) and those enrolled but not included in the analytic sample (n = 61) on pre-visit readiness, importance, confidence, discussion with the physician about alcohol consumption, provision of screening results to the physician, drinks per day, age, race, employment status, having a partner or drug use. There was a significant difference between study subjects and those enrolled but not included in the analytic sample on gender: subjects not included (i.e. lost to follow up) were more likely to be male (74% vs 58%, p = 0.05).

**Table 1:** Characteristics of 173 Adults with Unhealthy Alcohol Use Visiting a Primary Care Physician

**Characteristic**	**%(n)**
Female	42% (72)
*Race/ethnicity*	
African-American	58% (101)
White	18% (31)
Latino	16% (28)
Other	8% (13)
Employed (last 3 month)	60%(103)
Having a partner	68%(118)
Illicit drug use, last 30 days (any drug)	34% (58)
Patient's physician randomized to receive intervention (provision of screening results)	58% (100)
Discussion with physician about alcohol consumption	55% (95)
	**mean, (SD )**

Age	43.10 (12.61)
Drinks per day	3.05 (4.76)
Alcohol related consequences	8.41 (10.36)
*Readiness to change drinking measures (pre visit)*	
Readiness (1 to 10)	5.04 (3.13)
Importance (1 to 10)	6.01 (3.56)
Confidence (1 to 10)	7.75 (2.60)
RTCQ (-24 to 24)	3.32 (6.76)

Alcohol related consequences: Short Inventory of Problems score

RTCQ: Readiness To Change Questionnaire

Regarding generalizability, we compared included subjects to all other individuals identified by screening who were eligible. Subjects included in the analytic sample drank more (mean days drinking per week 2.98 vs 2.49, p = 0.01, mean number of drinks per typical day of consumption 4.45 vs 3.44, p < .0001). There were no significant differences (p < 0.05) between subjects included in the analytic sample and all individuals identified by screening on gender, race and readiness to change.

### Pre- to immediate post-visit changes

In unadjusted analyses of visual analog scales (VASs) (score range 1 to 10 points), subjects had significant increases immediately post-visit in readiness (+1.02 points, p < 0.0001), importance (+0.16, p < 0.0001), and confidence (+0.49, p = 0.003) when compared to pre-visit assessments (Table 2). Similarly, readiness as assessed by the RTCQ also increased (mean change +0.14, p < 0.0001). All of the differences indicated increases in readiness, importance, and confidence.

**Table 2:** Changes (from Before to) Immediately After a Primary Care Physician Visit in Patient "Readiness" to Change Drinking

**Measure (range)**	**Mean Change**	**(95% CI)**	**Effect size (d)**
**Readiness (1–10)**	1.02	(0.74, 1.30)	0.33
**Importance (1–10)**	0.16	(0.15, 0.18)	0.05
**Confidence (1–10)**	0.49	(0.16, 0.81)	0.19
**RTCQ (-24 - +24)**	0.14	(0.13, 0.15)	0.02

RTCQ: Readiness To Change Questionnaire

CI = confidence interval

In adjusted models (Table 3), a discussion with the physician about the patient's alcohol consumption was a significant positive predictor of an increase in readiness as measured by VAS (adjusted mean change +0.78 points, p = 0.04), as was not having a partner (+1.06, p = 0.006). Being white was a negative predictor of change in readiness (white versus other: -1.41, p = 0.002). Age, gender, alcohol consumption and alcohol-related problems were not significant predictors. Similarly for readiness measured by the RTCQ, discussion with the physician about the patient's alcohol consumption was a significant predictor of an increase (+1.14 points, p = 0.04). However, not having a partner was not significantly associated with an increase in RTCQ-measured readiness, and being white was associated with an increase (not a decrease) (+1.24, p = 0.03). None of the predictors were significantly associated with increases in importance and confidence.

**Table 3:** Predictors of Improvement Immediately After a Primary Care Physician Visit

**Improvement in**	**Readiness ** **(1–10)**		**Importance ** **(1–10)**		**Confidence ** **(1–10)**		**RTCQ ** **(-24- +24)**	
	**Mean adjusted change**	**(95% CI)**	**Mean adjusted change**	**(95% CI)**	**Mean adjusted change**	**(95% CI)**	**Mean adjusted change**	**(95% CI)**

Female	-0.37	(-1.19, 0.46)	0.21	(-0.34, 0.77)	-0.32	(-0.98, 0.34)	0.57	(-0.26, 1.41)
Age	0.01	(-0.02, 0.04)	-0.01	(-0.03, 0.02)	0.00	(-0.03, 0.03)	0.01	(-0.03, 0.04)
White	**-1.41**	(-2.31, -0.51)	-0.09	(-1.06, 0.89)	-0.36	(-0.86, 0.14)	**1.24**	(0.10, 2.39)
No Partner	**1.06**	(0.30, 1.81)	-0.05	(-0.65, 0.55)	-0.21	(-0.79, 0.37)	0.09	(-1.07, 1.25)
Drinks per day	0.02	(-0.08, 0.12)	0.04	(-0.10, 0.18)	-0.01	(-0.06, 0.05)	-0.03	(-0.18, 0.11)
Alcohol consequences (SIP score)	-0.03	(-0.06, 0.00)	-0.01	(-0.04, 0.01)	0.01	(-0.03, 0.05)	0.03	(-0.02, 0.07)
Discussion with physician	**0.78**	(0.05, 1.51)	0.16	(-0.46, 0.79)	-0.07	(-0.60, 0.46)	**1.14**	(0.04, 2.25)
Illicit drug use	-0.03	(-0.75, 0.68)	-0.51	(-1.27, 0.24)	0.07	(-0.52, 0.66)	0.87	(-0.41, 2.16)

SIP: Short Inventory of Problems

RTCQ: Readiness To Change Questionnaire

Bold entries have p-values < 0.05

### Pre-visit to 6-month follow up changes

Of the 173 subjects, 62 (36%) reported not drinking risky amounts during 30 days prior to the 6-month follow-up interview. But most subjects had improvements in drinking or readiness, importance and confidence: 62% were no longer drinking risky amounts or had improved readiness, 58% were not drinking risky amounts or had improved importance, and 56% were not drinking risky amounts or had improved confidence. Similarly, 67% of subjects were not drinking risky amounts or had improved RTCQ-readiness. We assessed the proportion of subjects who had improved because of a change in drinking. Of the 98 subjects who had improved drinking or readiness, 54 (55%) were no longer drinking risky amounts and 44 (45%) were still drinking risky amounts but had improved readiness. For improvement in drinking or importance, 62 (62%) had improved drinking, 38 (38%) improved importance; for improvement in drinking or confidence, 62 (64%) improved drinking, 35 (36%) confidence. Results were similar for improvement in drinking or RTCQ-readiness (62 (54%) had improved drinking, 53 (46%) improved RTCQ score.

In adjusted analyses, few predictors of improvement were identified. Not having a partner was a significant predictor of improvement in drinking or importance (p = 0.007), and drinking or confidence (p = 0.005). Being white was a negative predictor of improvement in drinking or readiness (p = 0.007). No other predictors were significantly associated with improvements in the hypothesized direction. Unexpectedly, having a discussion with the physician (at the initial visit) was a negative predictor (p = 0.03) of improvement in drinking or importance (Table 4).

**Table 4:** Predictors of Improvement 6 Months After a Primary Care Physician Visit

**Improvement in drinking or**...	**Readiness (1–10)**		**Importance (1–10)**		**Confidence (1–10)**		**RTCQ (-24- +24)**	
	**OR**	**(95% CI)**	**OR**	**(95% CI)**	**OR**	**(95% CI)**	**OR**	**(95% CI)**

Female	0.63	(0.30, 1.30)	0.60	(0.31, 1.18)	0.66	(0.32, 1.38)	0.91	(0.47, 1.73)
Age	1.02	(0.99, 1.05)	1.00	(0.97, 1.03)	1.01	(0.98, 1.04)	1.00	(0.98, 1.03)
White	**0.25**	(0.09, 0.69)	0.66	(0.29, 1.47)	0.47	(0.21, 1.03)	0.85	(0.36, 1.99)
No Partner	0.80	(0.41, 1.54)	**0.42**	(0.23, 0.79)	**0.44**	(0.25, 0.78)	0.30	(0.34, 1.99)
Drinks per day	0.98	(0.90, 1.07)	0.99	(0.93, 1.06)	0.98	(0.91, 1.06)	0.96	(0.89, 1.03)
Alcohol consequences (SIP score)	0.98	(0.95, 1.02)	1.00	(0.96, 1.04)	1.02	(0.98, 1.06)	0.99	(0.96, 1.03)
Discussion with physician	1.06	(0.58, 1.95)	**0.56**	(0.33, 0.95)	0.62	(0.27, 1.41)	0.85	(0.42, 1.73)
Illicit drug use	1.45	(0.65, 3.22)	0.71	(0.31, 1.63)	0.96	(0.42, 2.19)	1.61	(0.63, 4.09)

OR = odds ratio

SIP: Short Inventory of Problems

RTCQ: Readiness To Change Questionnaire

Bold entries have p-values < 0.05

## Discussion

We assessed variations in readiness, importance and confidence regarding changing drinking after a single primary care physician visit and improvements in these constructs and drinking 6 months later. After the visit, we observed significant increases in readiness, importance, and confidence. The effects were small (i.e. 1 point for readiness, 0.16 for importance, 0.49 for confidence, on a 1 to 10 scale). However, a clinically significant change in these constructs has not yet been well-defined, and the impact of changes of any magnitude is not known. Based on the transtheoretical model and motivational interviewing, clinicians are encouraged to help patients increase motivation, which in turn is expected to lead eventually to behavior change [10,35]. After a physician visit we can detect the beginning of such changes. Having a discussion about alcohol with the physician appeared to have an additional impact on readiness to change, an effect that was no longer detectable 6 months later. Like other measures of health states (e.g. blood pressure), "readiness to change" should be viewed as an instantaneous measure dependent on various internal and external influences.

In addition to these short-term changes, we studied the course of risky drinking and "readiness to change" in primary care patients. Six months after a physician visit, most subjects improved either their drinking or readiness. These improvements suggest that primary care physicians should be somewhat optimistic regarding the course of unhealthy alcohol use, with more than half of patients improving in a relatively short period of time.

We identified few predictors of changes in "readiness to change" and none that were consistent across measures or time. Not having a partner was a positive predictor of immediate changes in readiness but a negative predictor of improvement in two measures 6 months later. Being white was associated with worse readiness immediately after a physician visit and less improvement 6 months later but the finding was not confirmed in analyses with the RTCQ or the other two behavior change construct measures. Speculation regarding the mechanism for these hypothesis generating and inconsistent findings would be premature.

A discussion between the physician and the subject about alcohol predicted a positive change in readiness immediately after the visit (confirmed by the RTCQ), but paradoxically, was associated with less improvement in drinking or importance (but no change in VAS- or RTCQ-measured readiness). The fact that no association was found between discussion with the physician and drinking 6 months later may have been due to the use of ineffective counseling, but given the observed short-term effect, another explanation could be simply that this effect did not last.

Neither alcohol consumption, alcohol problems, nor illicit drug use significantly affected behavior change constructs or improvements. The fact that these markers of severity were not found to be negative predictors of improvement is of interest and should encourage physicians to address problems related to alcohol consumption even in the presence of concomitant illicit drug use, considering that most of their patients will have some improvement, independent of the severity of the alcohol problem.

A number of studies have assessed readiness to change and related constructs. In general, these studies have focused on characterizing specific populations [13,36-39] or on studying readiness as a predictor of behavior change [12,14]. Our study instead focused on how these constructs change over time. Improvement over time in untreated adults with unhealthy alcohol use and alcohol use disorders (alcohol abuse and dependence) has been previously reported [22,40,41]. Alcohol abuse and alcohol dependence seem to be (especially the latter) chronic conditions characterized by recurrent episodes of disease activity [42]. But the natural history of the spectrum of unhealthy alcohol use (risky drinking amounts through dependence) is not well described in the literature, nor is the natural history of readiness to change.

In addition to the aforementioned studies of the natural history of alcohol use disorders, studies of brief intervention for nondependent unhealthy alcohol use in primary care consistently report improvement over time in both treated and untreated individuals [43]. For example, male heavy drinkers in primary care decreased drinking over 3 years by 25 to 53% (depending on the outcome measure) in both intervention and control groups [44]. The improvement in drinking observed in our sample is in this range. Improvements such as these could be attributed to a regression to the mean [22]. However, our study sample was not primarily composed of very heavy drinkers, and we also observed improvements in readiness, importance and confidence regarding changing drinking, which were in the opposite direction than any hypothesized regression to the mean, given the relatively high levels of readiness, importance and confidence in the study population at baseline. Assessment effects (improvements due to being asked questions about drinking and discussing answers to those questions) have also been suggested as causes of improvements in drinking [45,46]. This exposure may have in part accounted for improvements in our sample. But if asking about alcohol and discussing drinking in primary care are in fact responsible for improvements, such effects should be viewed as favorable exposures in the primary care setting, and as part of the course in these patients, rather than as methodological nuisances.

This study has some strengths. To our knowledge, this is the first study to explore changes in readiness, importance and confidence during a single primary care visit. We described rapid changes in these constructs. We were also able to describe changes in readiness, importance or confidence and drinking over a 6 month period. Subjects studied were participants in a trial but there was no experimental brief counseling intervention nor a significant treatment effect on drinking amounts.

The study also has some limitations. First, the applicability of our findings may be limited to primary care patients with unhealthy alcohol use who agree to be screened and followed in a research study, and to those with similar characteristics as in our sample (e.g. 32% reporting no alcohol problems, a third with illicit drug use). Although participants differed little from those who did not participate, participants did drink more. Similarly, subjects lost to the analytic sample differed little from those studied except on gender. The effects of these differences can be considered in the interpretation of our results. Second, findings are from secondary data analyses. Causality (of predictors) cannot be inferred, and there could be many explanations for changes in the readiness constructs. However, observational studies such as this one are likely among the best ways to study the natural course of behavior change constructs and changes in drinking, particularly prospective studies. Third, in our attempt to explore changes over time, we had to combine actual behavior (changes in alcohol consumption) with cognitions about behavior change. It is likely that these two dimensions reflect different aspects of behavior. We presented data on the dimensions separately (and combined) but performed regression analyses on the combined outcome. From a clinical perspective, improvements in either drinking or readiness (combined) seem to be most relevant. Also, the interpretation of a 6 month change in readiness on a continuous scale for someone who continues to drink risky amounts is difficult, since it not clear how one would interpret a change in some number of points. Fourth, we did not adjust the level of significance for multiple comparisons. As such one should be cautious about interpreting associations, particularly those not in the hypothesized direction. Lastly, all data were obtained from interviews and are subject to recall and social desirability biases. But interviews with trained research associates and assurances of confidentiality took place immediately after the primary care visit to maximize accurate recall and minimize bias. Nonetheless, we do not know what patients meant by discussions about alcohol, which could have been brief or extensive and may or may not have included known effective components of brief interventions.

## Conclusion

Our results provide important information. First, subjects appear to change readiness, importance and confidence after a single physician visit. Second, most patients with unhealthy alcohol use will improve 6 months after a primary care visit, either on behavioral change constructs or drinking. Third, factors usually associated with worse alcohol treatment outcomes (e.g. drug use, alcohol related problems) do not seem to prevent improvement. Future research should focus on specific measures of behavior change constructs, perhaps assessed by ecological momentary assessments of these rapidly changing dimensions [47,48] and what contributes to their changing, and how and when they contribute to actual behavior change. A better understanding of these mechanisms could be used to enhance interventions for unhealthy alcohol use in primary care settings, which at present, are only modestly effective. But even if the reasons for the improvements in drinking and behavior change constructs are mostly unknown, primary care physicians should be aware of the prognosis for patients with unhealthy alcohol use, to recognize incremental steps towards change and to support their patient's efforts.

## Competing interests

The authors declare that they have no competing interests.

## Authors' contributions

NB, NH, and RS conceived and designed the study. RS led implementation of the SIP study randomized trial. NH led and programmed the statistical analyses. NB wrote the first draft of the manuscript. All authors read and approved the final manuscript.

## Pre-publication history

The pre-publication history for this paper can be accessed here:



## References

[B1] Saitz R (2005). Clinical practice. Unhealthy alcohol use. N Engl J Med.

[B2] Rehm J, Room R, Monteiro M (2003). Alcohol as a risk factor for global burden of disease. Eur Addict Res.

[B3] Bertholet N, Daeppen JB, Wietlisbach V, Fleming M, Burnand B (2005). Reduction of alcohol consumption by brief alcohol intervention in primary care: systematic review and meta-analysis. Arch Intern Med.

[B4] Bien TH, Miller WR, Tonigan JS (1993). Brief interventions for alcohol problems: a review. Addiction.

[B5] Dunn CW, Ries R (1997). Linking substance abuse services with general medical care: integrated, brief interventions with hospitalized patients. Am J Drug Alcohol Abuse.

[B6] Rollnick S, Miller WB, Heather N (1998). Readiness, Importance, and Confidence. Treating Addictive Behaviors.

[B7] Samet JH, Rollnick S, Barnes H (1996). Beyond CAGE. A brief clinical approach after detection of substance abuse. Arch Intern Med.

[B8] Barnes HN, Samet JH (1997). Brief interventions with substance-abusing patients. Med Clin North Am.

[B9] Prochaska JO, DiClemente CC, Norcross JC (1992). In search of how people change: Applications to addictive behaviors. Am Psychol.

[B10] Miller WR, Wilbourne PL (2002). Mesa Grande: a methodological analysis of clinical trials of treatments for alcohol use disorders. Addiction.

[B11] Bandura A (1977). Social Learning Theory.

[B12] Demmel R, Beck B, Richter D, Reker T (2004). Readiness to change in a clinical sample of problem drinkers: relation to alcohol use, self-efficacy, and treatment outcome. Eur Addict Res.

[B13] Williams EC, Kivlahan DR, Saitz R (2006). Readiness to change in primary care patients who screened positive for alcohol misuse. Ann Fam Med.

[B14] Maisto SA, Conigliaro J, McNeil M, Kraemer K, O'Connor M, Kelley ME (1999). Factor structure of the SOCRATES in a sample of primary care patients. Addict Behav.

[B15] Heather N, Rollnick S, Bell A (1993). Predictive validity of the Readiness to Change Questionnaire. Addiction.

[B16] Maisto SA, Carey KB, Bradizza CM, Leonard KE, Blane HT (1999). Social learning theory. Psychological Theories of Drinking and Alcoholism.

[B17] Williams EC, Horton NJ, Samet JH, Saitz R (2007). Do brief measures of readiness to change predict alcohol consumption and consequences in primary care patients with unhealthy alcohol use?. Alcohol Clin Exp Res.

[B18] Forsberg L, Ekman S, Halldin J, Ronnberg S (2004). The readiness to change questionnaire: reliability and validity of a Swedish version and a comparison of scoring methods. Br J Health Psychol.

[B19] Project MATCH Research Group (1997). Matching Alcoholism Treatments to Client Heterogeneity: Project MATCH posttreatment drinking outcomes. J Stud Alcohol.

[B20] Anton RF, O'Malley SS, Ciraulo DA (2006). Combined pharmacotherapies and behavioral interventions for alcohol dependence: the COMBINE study: a randomized controlled trial. Jama.

[B21] Wampold BE, Mondin GW, Moody M, Stich F, Benson K, Ahn H (1997). A Meta-Analysis of Outcome Studies Comparing Bona Fide Psychotherapies: Empirically, "All Must Have Prizes". Psychological Bulletin.

[B22] Moyer A, Finney JW (2002). Outcomes for untreated individuals involved in randomized trials of alcohol treatment. J Subst Abuse Treat.

[B23] Saitz R, Svikis D, D'Onofrio G, Kraemer KL, Perl H (2006). Challenges applying alcohol brief intervention in diverse practice settings: populations, outcomes, and costs. Alcohol Clin Exp Res.

[B24] Saitz R, Horton NJ, Sullivan LM, Moskowitz MA, Samet JH (2003). Addressing alcohol problems in primary care: a cluster randomized, controlled trial of a systems intervention. The screening and intervention in primary care (SIP) study. Ann Intern Med.

[B25] Mayfield D, McLeod G, Hall P (1974). The CAGE questionnaire: validation of a new alcoholism screening instrument. Am J Psychiatry.

[B26] National Institute on Alcohol Abuse and Alcoholism (1995). The Physicians' Guide to Helping Patients with Alcohol Problems.

[B27] Sobell LC, Sobell MB, Litten RZAJ (1992). Timeline follow-back: a technique for assessing self reported alcohol consumption. Measuring Alcohol Consumption: Psychosocial and Biochemical Methods.

[B28] Miller WR, Rollnick S (2002). Motivational Interviewing: Preparing People for Change.

[B29] Rollnick S, Heather N, Gold R, Hall W (1992). Development of a short 'readiness to change' questionnaire for use in brief, opportunistic interventions among excessive drinkers. Br J Addict.

[B30] Budd R, Rollnick S (1996). The structure of the readiness to change questionnaire: a test of Prochaska & DiClemente's transtheoretical model. British Journal of Health Psychology.

[B31] McNally AM, Palfai TP (2001). Negative emotional expectancies and readiness to change among college student binge drinkers. Addict Behav.

[B32] Sobell LC, Brown J, Leo GI, Sobell MB (1996). The reliability of the Alcohol Timeline Followback when administered by telephone and by computer. Drug Alcohol Depend.

[B33] Miller WR, Tonigan J, Longabaugh R (1995). The Drinker Inventory of Consequences (DrInC). An Instrument for Assessing Adverse Consequences of Alcohol Abuse Test Manual.

[B34] Freyer-Adam J, Coder B, Baumeister SE (2008). Brief alcohol intervention for general hospital inpatients: A randomized controlled trial. Drug Alcohol Depend.

[B35] DiClemente CC, Schlundt D, Gemmell L (2004). Readiness and stages of change in addiction treatment. Am J Addict.

[B36] Vik PW, Culbertson KA, Sellers K (2000). Readiness to change drinking among heavy-drinking college students. J Stud Alcohol.

[B37] Apodaca TR, Schermer CR (2003). Readiness to change alcohol use after trauma. J Trauma.

[B38] Bombardier CH, Ehde D, Kilmer J (1997). Readiness to change alcohol drinking habits after traumatic brain injury. Arch Phys Med Rehabil.

[B39] Rumpf HJ, Hapke U, Meyer C, John U (1999). Motivation to change drinking behavior: comparison of alcohol-dependent individuals in a general hospital and a general population sample. Gen Hosp Psychiatry.

[B40] Sobell LC, Ellingstad TP, Sobell MB (2000). Natural recovery from alcohol and drug problems: methodological review of the research with suggestions for future directions. Addiction.

[B41] Bischof G, Rumpf HJ, Hapke U, Meyer C, John U (2003). Types of natural recovery from alcohol dependence: a cluster analytic approach. Addiction.

[B42] Venner KL, Matzger H, Forcehimes AA (2006). Course of recovery from alcoholism. Alcohol Clin Exp Res.

[B43] Bertholet N, Schwan R, Duhamel O, Perney P, Daeppen JB (2003). Efficacité de l'intervention brève. Alcoologie et Addictologie.

[B44] Aalto M, Sillanaukee P (2000). Compliance rate and associated factors for entering an alcohol brief intervention treatment programme. Alcohol Alcohol.

[B45] Clifford PR, Maisto SA, Davis CM (2007). Alcohol treatment research assessment exposure subject reactivity effects: part I. Alcohol use and related consequences. J Stud Alcohol Drugs.

[B46] Maisto SA, Clifford PR, Davis CM (2007). Alcohol treatment research assessment exposure subject reactivity effects: part II. Treatment engagement and involvement. J Stud Alcohol Drugs.

[B47] Shiffman S, Stone AA, Hufford MR (2008). Ecological momentary assessment. Annu Rev Clin Psychol.

[B48] Hufford MR, Shields AL, Shiffman S, Paty J, Balabanis M (2002). Reactivity to ecological momentary assessment: an example using undergraduate problem drinkers. Psychol Addict Behav.

